# Effects of dietary supplementation of organic and inorganic zinc on the performance characteristics, tissue mineralization, apparent mineral retention, and antioxidant status of broiler chicks

**DOI:** 10.1186/s12917-025-04976-6

**Published:** 2025-08-28

**Authors:** Bruno Reis de Carvalho, Pedro Righetti Arnaut, Jorge Cunha Lima Muniz, Hélvio da Cruz Ferreira, Nathana Rudio Furlani, Warley Júnior Alves, James Eugene Pettigrew, Melissa Izabel Hannas, Gabriel da Silva Viana

**Affiliations:** 1 Department of Animal Science, Federal University of Viçosa, Viçosa, 36570-900 Minas Gerais Brazil; 2 Pettigrew Research Services US, Champaign, IL USA; 3 Production Systems, Natural Resources Institute Finland (Luke), Jokioinen, Finland

**Keywords:** Antioxidant defense, Mineral retention, Trace mineral requirement

## Abstract

A 10-day study was carried out to evaluated the effects of organic (zinc proteinate, ZnPro) and inorganic (zinc sulfate, ZnSO₄•H₂O) zinc supplementation on growth, tissue mineralization, mineral excretion, and antioxidant responses in broiler chickens. Male Cobb 500 chicks were allotted into 10 treatments, and ten replicates per treatment with five birds per replicate in a 2 × 5 factorial design. Dietary Zn was supplied at 0, 19, 38, 57, and 76 mg/kg. ZnPro feeds included proteinates for copper, iron, and manganese, while Zn-sulfate diets used inorganic salts.

**Results **Body weight (BW) and average daily gain (ADG) improved at 19 mg Zn/kg for both sources. Feed conversion ratio (FCR) was optimized at 19 mg Zn/kg for ZnPro and 30 mg Zn/kg for Zn-sulfate. Interactive effect was observed for Zn intake, excretion and retention. At higher inclusion of ZnPro levels (≥ 38 mg Zn/kg), chicks showed increased Zn intake and excretion. ZnPro levels higher than ≥ 56 mg Zn/kg promoted higher Zn retention. Zn retention in liver and tibia were maximized at 38 mg Zn/kg for both sources, and were similar until 76 mg Zn/kg. ZnPro resulted in higher liver Zn concentration and greater tibia manganese (Mn) deposition, while Zn-sulfate increased liver iron (Fe) concentration. Additionally, Zn-sulfate enhanced glutathione peroxidase (GSH-Px) activity in liver and breast muscle, while both sources modulated liver superoxide dismutase (SOD) activity.

**Conclusions **Both Zn sources supported growth and antioxidant responses, with optimal DWG levels at 30 mg Zn/kg for Zn-sulfate and 19 mg Zn/kg for ZnPro, highlighting greater efficiency of the organic source. Inorganic Zn further boosted GSH-Px activity, enhancing tissue antioxidant capacity, while ZnPro demonstrated advantages in tissue mineral retention. Further research on environmental impacts of Zn excretion from high ZnPro diets is recommended.

## Background

Zinc (Zn) is an essential element for poultry survival, health, growth, and reproduction. As cofactor for more than 300 metalloenzymes, Zn participates in the synthesis of structural proteins such as collagen and keratin, which form cartilage, bone, feather, skin, beak, and claws [1, 2]. Furthermore, Zn serves as cofactor of CuZn-superoxide dismutase (SOD), a metalloenzyme found in the cytosol, nucleus, peroxisomes, and mitochondria of eukaryotic cells [3]. CuZn-superoxide dismutase protects cells from the toxicity of free superoxide radicals, whose effect on phospholipid membranes, proteins in the cytosol and DNA are associated with intracellular damage and apoptosis [4]. Signs of Zn deficiency in broilers include impaired growth and feed efficiency, immune and antioxidant defense suppression, poor bone mineralization as either osteopenia or osteoporosis, and skeletal malformation [5, 6]. On the other hand, excessive dietary Zn supply has been shown to be detrimental for growth and feed efficiency by inhibiting intestinal absorption of other minerals such as Cu, inducing its deficiency [7]. That could affect, in turn, the transport and metabolism of iron (Fe) in different tissues of the body [8].

Zinc has been conventionally supplied in poultry feeds in its inorganic form, but in the last two decades broiler responses to organic Zn sources became a topic of great interest and discussion. Although published outcomes generally confirm superior bioavailability of organic Zn compared with inorganic salts, the requirement of Zn for broilers remains unclear. The National Research Council [9] estimated broiler requirements for Zn as 40 mg/kg diet based on the studies of [10–12] in which semi purified diets were supplemented with Zn sulphates. Zinc is involved in the metabolism of several molecules, and broiler metabolic rates have been dramatically changed by genetic selection over the years [13]. Features of modern genotypes include higher metabolic rates, higher protein accretion rates in body and feathers, and consequently a higher susceptibility to oxidative stress due to intense cellular metabolism. As many of such processes are regulated by Zn metalloenzymes, it seems reasonable to hypothesize that broiler requirements for Zn might be quite different from those established 50 years ago. Therefore, this study explored the response of broiler chickens to dietary Zn concentrations and sources. And based on such responses, determined the Zn requirements for both sources.

## Results

### Growth performance, Zn retention and requirements

The analyzed concentrations of Zn in the test diets were 15, 34, 48, 66 and 72 mg Zn/kg in Zn-sulfate diets, and 15, 33, 53, 72 and 85 mg Zn/kg in ZnPro diets (Table 1). Overall, such values are similar to those expected when adding 0, 19, 38, 57 and 76 mg Zn/kg to a basal diet containing 15 mg Zn/kg. As shown in Table 2, interactive effects between sources and levels were observed for the BW, ADG and ADFI (*P* < 0.05) of chicks Graded Zn supplementation influenced quadratically (*P* < 0.05) BW, ADG and ADFI for both sources. At 19 mg added Zn/kg diet, chicks fed ZnPro diets exhibited greater ADG compared with Zn-sulfate fed group (*P* < 0.05). Significant difference was observed (*P* < 0.005) At 38 and 57 mg inclusion of Zn/kg diet BW and ADG of Zn-sulfate dietary treatments fed chicks were greater compared with chicks fed ZnPro diets. Body weight and ADG of chicks fed Zn-sulfate supplemented diets were lowest (*P* < 0.05) on the basal diet and indicating similarity among the other levels. Birds fed ZnPro diets exhibited greater (*P* < 0.05) BW and ADG at 19 mg Zn/kg compared to the basal diet, but growth was lower (*P* < 0.05) with higher dietary Zn levels. At 38 and 76 mg Zn/kg diet, chicks fed ZnPro diets exhibited a lower ADFI (*P* < 0.05) compared with chicks fed diets containing Zn-sulfate. Feed conversion ratio was quadratically influenced (*P* < 0.05) as Zn supplementation increased. Chicks fed the basal diets exhibited the worst FCR (*P* < 0.05) among treatment groups. The interaction effect of Zn sources and levels on Zn intake, excretion and apparent mineral retention in broiler chickens (Table 3) revealed that Zn intake, Zn retention and Zn excretion were significantly (*P* < 0.05) influenced by Zn sources and levels.

**Table 1:** Analyzed supplemented levels of zinc and trace minerals in experimental diets, as fed basis

Mineral sources^1^	Trace minerals^2^	Zn supplementation in semi-purified basal diet^1^ (mg/kg)				
		**0**	**19**	**38**	**57**	**76**
Organic	Zn	15	33	53	72	85
	Cu	12	12	12	12	12
	Fe	102	102	102	106	100
	Mn	102	99	101	100	95
Inorganic	Zn	15	34	48	66	72
	Cu	10	10	10	10	10
	Fe	156	159	153	146	165
	Mn	64	66	67	62	59

^1^Zn supplementation levels were obtained by adding: 0, 19, 38, 57, and 76 mg Zn/kg diet to a semi-purified basal diet containing 15 mg Zn/kg

^2^Value determined by analysis. Each value based on 5 replicates

**Table 2:** Growth performance of broiler chicks fed different dietary supplementation levels of zinc provided by organic and inorganic sources for broilers from 8 to 17 d of age

Item		BW, g/bird			ADG, g/bird/day			ADFI, g/bird/day			FCR, g/g
Zn level		Inorganic	Organic		Inorganic	Organic		Inorganic	Organic		Levels
0		452 B	459 C		31.3 B	32.1 C		45.7 B	46.9 B		1.46 A
19		485 A	494 A		34.7 Ab	36.1 Aa		48.9 A	49.8 A		1.40 B
38		487 Aa	474 Bb		35.2 Aa	33.8 Bb		49.5 Aa	47.8 ABb		1.41 B
57		490 Aa	480 Bb		35.6 Aa	34.5 Bb		49.5 A	48.2 AB		1.34 B
76		482 A	480 B		34.7 A	34.4 B		49.1 Aa	47.5 Bb		1.40 B
Average		480	477		34.3	34.2		48.5	48.0		1.40
SEM^1^	11.6			1.22			1.68			0.049	
S*L^2^	<0.01			<0.01			0.013			0.361	
Source	0.387			0.539			0.132			0.313	
L^3^		<0.01	0.019		<0.01	0.015		<0.01	0.902		<0.01
Q^4^		<0.01	<0.01		<0.01	<0.01		<0.01	0.018		<0.01

^A-B-C, a-b^Different uppercase letters in the same column and lowercase letters in the same line for each parameters are different by Tukey test at 5%

^1^Standard error of means

^2^Interactive effects between zinc sources and levels

^3^Linear effect of supplementation zinc levels in each source

^4^Quadratic effect of supplementation zinc levels in each source

**Table 3:** Zinc retention of broiler chicks fed different dietary supplementation levels of zinc provided by organic and inorganic sources

**Item**		Intake, g/bird/day			Excreted, g/bird/day			Retention, g/bird/day			Apparent Zn Retention, %
**Zn level **		Inorganic	Organic		Inorganic	Organic		Inorganic	Organic		Levels
0		0.586 E	0.587 E		0.322 D	0.292 E		0.263 C	0.296 C		49.0 A
19		1.37 D	1.32 D		0.744 C	0.723 D		0.625 B	0.592 B		45.2 A
38		1.99 Cb	2.17 Ca		1.06 Bb	1.24 Ca		0.924 A	0.930 A		44.6 A
57		2.69 Bb	2.93 Ba		1.79 A	1.76 B		0.902 Ab	1.11 Aa		36.6 B
76		2.98 Ab	3.39 Aa		1.93 Ab	2.24 Aa		0.989 Ab	1.14 Aa		33.7 B
Average		1.91	2.08		1.17	1.25		0.741	0.825		41.8
SEM^1^	0.149			0.120			0.0362			0.82	
*P*-value											
Source	<0.01			0.012			0.022			0.363	
S*L^2^	<0.01			<0.01			0.022			0.363	
L^3^		<0.01	<0.01		<0.01	<0.01		<0.01	<0.01		<0.01
Q^4^		<0.01	<0.01		0.394	0.592		<0.01	<0.01		0.187

^A-B-C-D-E, a-b^Different uppercase letters in the same column and lowercase letters in the line for each parameter are different by Tukey test at 5%

^1^Standard error of means

^2^Interactive effects between zinc sources and levels

^3^Linear effect of supplementation zinc levels in each source

^4^Quadratic effect of supplementation zinc levels in each source

Chicks fed diet supplemented Zn levels equivalent or higher than 38 mg/kg diet from ZnPro exhibited a higher intake and excretion of Zn (*P* < 0.05) compared with Zn-sulfate fed chicks, except at the level of 57 mg/kg where no significant differences (*P* > 0.05) between sources were observed for Zn excretion. The highest excretion of Zn (*P* < 0.05) was at the levels of 57 and 76 mg Zn/kg ZnPro inclusion diets, and at 76 mg Zn/kg Zn-sulfate supplemented diets. For both Zn sources, maximum apparent Zn retention was achieved at 38 mg Zn/kg diet (*P* < 0.05); however, at 57 and 76 mg Zn/kg diet, Zn retention in ZnPro fed chicks was higher (*P* < 0.05) compared with Zn-sulfate.

Estimates of chick requirements for Zn for optimal growth according to a CL (curvilinear plateau) regression model are detailed in Table 6. Body weight, ADG and FCR were optimized at 22.67, 30.32 and 29.20 mg Zn/kg in Zn-sulfate supplemented diets, respectively. In ZnPro diets, BW, ADG, achieved the greatest values at 13.6 and 18.7 mg Zn/kg, respectively, and FCR was best at 19.0 mg Zn/kg.

### Zinc tissue concentration and antioxidant enzyme activit

As detailed in Table 4, no interactions between Zn sources and levels were observed in Zn concentration in breast muscle, liver, and tibia (*P* > 0.05). Breast muscle Zn content tended (*P* = 0.06) to rise linearly with Zn supplementation. Zinc supplementation elicited a quadratic response in the concentration of Zn in chick liver and tibia (*P* < 0.05). Zinc concentration in liver and tibia achieved the greatest value (*P* < 0.05) in chicks fed 38 mg Zn/kg supplemented diet and remained constant until the highest level under study (76 mg Zn/kg diet). The Zn concentration in the liver of chicks fed diets with ZnPro supplementation was higher (*P* < 0.05) compared with those fed diets with Zn-sulfate supplementation. A tendency for interactive effects (*P* = 0.073) between Zn sources and supplemental levels were noticed on the Mn concentration in the liver and tibia, respectively. ZnPro quadratically influenced (*P* < 0.05) the Mn deposition in the tibia and the greatest deposition was noticed at the level of 76 mg Zn/kg diet. ZnPro supplementation promoted a higher (*P* < 0.05) concentration of Mn in chick liver compared to Zn-sulfate. Chicks fed Zn-sulfate, however, had higher (*P* < 0.05) Fe liver concentration compared with chicks fed ZnPro supplemented diet. Graded Zn supplementation influenced quadratically (*P* < 0.05) and tended to influence linearly (*P* = 0.078) the Mn and Fe concentration in the liver and tibia, respectively.

**Table 4:** Mineral concentration (dry matter) on tissues of broiler chicks fed different dietary supplementation levels of zinc provided by organic and inorganic sources

**Item**	**Zinc levels (mg/kg)**					**Source**		SEM^1^	**Source**	**Level**	S*L^2^	L^3^	Q^4^
	**0**	**19**	**38**	**57**	**76**	**Inorganic**	**Organic**						
*Breast muscle*													
Zinc, mg/kg	19.5	20.1	21.2	20.6	21.0	20.7	20.3	0.20	0.297	0.298	0.405	0.054	0.846
Copper, mg/kg	1.85	1.82	1.82	1.88	1.77	1.84	1.82	0.035	0.792	0.902	0.691	0.660	0.729
Manganese, mg/kg	0.587	0.632	0.610	0.644	0.665	0.670	0.585	0.024	0.114	0.897	0.453	0.341	0.967
Iron, mg/kg	27.8	28.5	27.1	26.8	28.3	28.3	27.2	0.66	0.431	0.925	0.631	0.912	0.639
Calcium, g/kg	120	109	113	113	109	110	116	0.0	0.270	0.604	0.960	0.286	0.692
Phosphorus, g/kg	8.93	8.90	9.27	9.22	9.03	8.90	9.24	0.095	0.054	0.544	0.987	0.394	0.306
*Liver*													
Zinc, mg/kg	67.6 C	75.2 B	79.8 A	85.0 A	82.6 A	75.9	80.2	0.89	<0.01	<0.01	0.382	<0.01	<0.01
Copper, mg/kg	14.0	12.8	12.6	12.8	12.7	13.2	12.8	0.22	0.414	0.370	0.601	0.114	0.180
Manganese, mg/kg	9.77	9.02	9.04	9.27	10.0	8.66	10.2	0.16	<0.01	0.089	0.516	0.459	0.007
Iron, mg/kg	397	373	308	351	337	410	295	14.0	<0.01	0.216	0.177	0.112	0.228
Calcium, g/kg	166	158	155	166	153	161	158	0.0	0.449	0.086	0.073	0.177	0.755
Phosphorus, g/kg	11.4	11.5	11.3	11.5	11.3	11.4	11.4	0.09	0.683	0.911	0.769	0.712	0.822
*Tibia*													
Zinc, mg/kg	52 C	137 B	150 A	169 A	185 A	136	142	5.1	0.214	<0.01	0.395	<0.01	<0.01
Copper, mg/kg	3.44	3.42	3.49	3.53	3.29	3.40	3.47	0.044	0.405	0.455	0.285	0.555	0.192
Manganese, mg/kg	5.82	5.56	5.39	5.58	5.90	5.16	6.14	0.115	<0.01	0.497	0.015	0.805	0.074
Iron, mg/kg	171	187	194	187	195	189	185	3.8	0.666	0.283	0.467	0.078	0.365
Calcium, g/kg	141	155	156	148	151	151	150	2.5	0.862	0.352	0.592	0.448	0.140
Phosphorus, g/kg	74.8	76.3	76.1	75.5	77.2	76.1	75.9	0.52	0.880	0.638	0.447	0.298	0.968

^A-B-C^Different uppercase letters in the same line are different by Tukey test at 5%

^1^Standard error of means

^2^Interactive effects between zinc sources and levels

^3^Linear effect of supplementation zinc levels

^4^Quadratic effect of supplementation zinc levels

No interactive effects were observed between levels and sources of Zn (*P* > 0.05) in the SOD and GSH-Px activities in tissues (Table 5). Graded Zn supplementation influenced quadratically (*P* < 0.05) the SOD activity in chick liver. Chicks fed Zn-sulfate supplemented diets exhibited higher activity of GSH-Px in breast muscle and liver (*P* < 0.05) compared with ZnPro fed chicks.

**Table 5:** Antioxidant enzyme activity on breast muscle and liver of broiler chicks fed different dietary supplementation levels of zinc provided by organic and inorganic sources

Item	**Zinc levels (mg/kg)**					**Sources**		SEM^1^	**Source**	**Level**	S*L	L^2^	Q^3^
	**0**	**19**	**38**	**57**	**76**	**Inorganic**	**Organic**						
*Liver*													
Superoxide dismutase (g/g pro)	53.3	56.9	60.2	57.2	56.8	58.0	55.9	0.81	0.200	0.139	0.867	0.209	0.040
Glutathione peroxidase (IU/g pro)	990	1092	1096	1108	1074	1315	828	51.3	<0.01	0.935	0.791	0.579	0.501
*Breast muscle*													
Superoxide dismutase (g/g pro)	5.25	5.29	5.47	5.56	5.54	5.40	5.45	0.097	0.822	0.806	0.995	0.234	0.807
Glutathione peroxidase (IU/g pro)	15984	18173	17164	15545	17724	21149	12687	618	<0.01	0.293	0.477	0.789	0.867

^1^Standard error of means for treatments

^2^Interactive effects between zinc sources and levels

^2^Interactive effects between zinc sources and levels

^4^Quadratic effect of supplementation zinc levels in each source

## Discussion

The current study was designed to measure the effects of dietary supplementation of Zn sources and levels on the performance, mineral excretion and balance, tissue mineralization and antioxidant responses in broiler chickens. Additionally, regression models were fitted to determine organic and inorganic Zn supplemental levels which improved the broiler performance. Expectedly, irrespective of the sources investigated, broiler performance improved when dietary Zn supplementation was provided, which demonstrated the essentiality of this trace mineral for the growth of the bird. Except for FCR, interactive effects between Zn sources and levels were found on all the performance measures assessed. In both Zn sources, ADG and BW responses reached the maximum values at the supplemental level of 19 mg Zn/kg, which corresponds to the dietary Zn concentration of 34 mg/kg. Similar results were reported by [14] who observed that the dietary supplementation of Zn dose of 10 mg/kg as Zn proteinate or Zn sulphate in corn and soybean-based diets containing 23 mg Zn/kg stabilized broiler ADG responses, suggesting that 33 mg Zn/kg feed met broiler nutritional needs for growth. In chicks fed Zn-sulfate supplemented diets, both responses were stabilized at 19 mg supplemental Zn/kg and remained constant up to the highest supplemental level under study. Conversely, both ADG and BW of chicks fed ZnPro diet were slightly impaired at the supplemental levels of 38, 57, and 76 mg Zn/kg. The result obtained in this study contradicted the findings on the recommended Zn level of 40 mg/kg described by [9] who supported an adequate growth rates of fast-growing broiler genotypes currently utilized in intensive poultry rearing. The outcomes reported herein are in line with those published by [15–17] that the dietary Zn concentration equivalent to 35 mg/kg in cereal based diets met the nutritional demands of broiler chickens for growth. Similarly [18], reported that supplemental Zn doses in feeds containing 30 mg Zn/kg provided solely by cereal-based feeds did not elicit any additional increment in the growth of broilers. However, it is noted that despite the similarity between the findings of this study and the referred estimates on the Zn concentration as reported by the authors are sufficient to support the growth (30–35 mg/kg) provided by the cereal-based diets used in the assay without supplementation of Zn sources.

Another conclusion that could be drawn from the performance responses observed in this study is that the tolerance of chicks to doses of Zn that exceeded the requirements for growth is lower for organic sources compared with inorganic. At the supplemental levels of 38 and 57 mg Zn/kg chick ADG was slightly impaired in birds fed ZnPro compared with those that were s fed Zn-sulfate supplemented feeds. The higher bioavailability of ZnPro compared with Zn-sulfate can be clearly demonstrated by our findings on mineral retention. Birds fed 57 and 76 mg Zn/kg supplemented with ZnPro exhibited a higher Zn retention compared with that observed in birds fed Zn-sulfate feeds, this suggested a superior absorption and accumulation in tissues of the birds from provided by the organic Zn source. The fraction of the absorbed Zn intake, Zn retention in percentage, remained unaffected up to the supplemental level of 38 mg Zn/kg (53 mg Zn/kg feed), irrespective of the sources under study, but was 18 and 25% lower at the supplemental levels of 57 and 76 mg/kg, respectively. Such responses indicated supplementation at higher doses Zn absorption was compromised. It was noticed that the concentration of ZnPro in the liver of birds fed Zn supplemented diets was 5% higher than that in birds fed Zn-sulfate supplemented diets. The outcomes also showed that the inclusion of Zn levels influenced Zn deposition in the liver and tibia. Zinc concentration in both tissues rose with the increase in Zn level to 38 mg/kg and stabilized at higher levels. Though there was a linear rise in Zn deposition in the breast muscle because of Zn supplementation, Zn concentration in breast muscle remained unaffected by the dietary Zn doses and sources as investigated in the study. These outcomes supported the report of [19]. Such findings suggested that Zn accumulated preferentially in bones and liver, being excreted when the storage capacity of such organs is exceeded. It was also indicated in literature that the pancreas is another organ in which Zn is usually stored when provided at high concentrations in poultry feeds [20].

In the current study, Zn dietary sources also affected the Fe and Mn concentration in tibia and liver. Chicks fed ZnPro supplemented feeds exhibited a higher concentration of Zn (5%) and Mn (15%) in liver compared with birds fed diets containing Zn-sulfate. Although the postabsorptive metabolism of trace minerals and their interactions in poultry are too complex to be entirely illustrated and understood, literature suggested a possible synergy between Zn and Mn uptake by liver tissue [21]. Evidence indicates that Zn transporters can also be responsible for the uptake of Mn into cells, and in the case of liver, particularly, the transporter ZIP14 is the one responsible for Zn uptake to cells [22]. In the current study, it is possible that ZnPro might have regulated the expression of Zn transporters in liver, which consequently might have also favored not only Zn, but also Mn uptake into hepatocytes. It was highlighted in the current study that ZnPro was supplied in combination with other trace minerals in organic form including Mn whose bioavailability is higher than Mn sulphate provided in Zn-sulfate [23]. Therefore, in ZnPro supplemented diets, the potential agonistic interactions among trace minerals in intestinal lumen might have been minimized, which consequently allowed greater absorption of Mn, and other trace minerals. Although not necessarily required, the divalent metal-ion transporter-1 (DMT-1), the main transporter of Fe, might be also involved in the intestinal absorption of Mn at the apical membrane of enterocytes [22]. When recognized and absorbed as peptides and/or amino acids, the competition between these trace minerals could be minimized leading to an increase of their concentration in different organs. The liver plays a primary role in the homeostasis of Zn and other trace minerals as it stores such elements from portal blood and regulates their delivery to different tissues and their excretion through endogenous intestinal losses [24, 25]. Beyond Mn, Fe metabolism was also affected by the Zn sources as observed in the study. Nonetheless, contrary to the former element, the latter had a lower liver concentration with the administration of ZnPro. The transport of post absorbed dietary Fe stored in the liver to extra hepatic tissues is highly regulated by ceruloplasmin (CP), a Cu-dependent enzyme whose serum concentration in broilers has shown to be positively modulated by organic sources of Cu compared with inorganic Cu sulfate [26] [27]. has demonstrated that Cu deficiency caused Fe accumulation in the liver of rodents, and [28] noticed that dietary supplemental Cu linearly increased Fe concentration in the liver of broiler chickens, and that birds fed Cu proteinate stored 20% less Fe in liver compared with birds fed Cu sulfate (CuSO_4•_5H_2_O). Presumably, a higher absorption of Cu proteinase supplemented in ZnPro feeds might have also improved the Fe mobilization in the liver of the birds.

The bones are the main mineral storage tissue in the body. The fast growth rates of the modern broiler strains increase the susceptibility of birds to develop leg abnormalities that might impair the proper development of limbs, reducing voluntary feed intake and meat production [29]. Zinc plays a crucial role in bone development by influencing collagen synthesis, osteocalcin, and alkaline phosphatase production, which stimulate the calcium deposition in the bones [30–33]. Literature has shown that the dietary supplementation of organic Zn increases bioavailability of other trace minerals [29, 33]. The greater mineral deposition in the tibia is directly related to its strength and resistance [33, 34]. In our study, Zn deposition in the tibia was only influenced by the dietary Zn levels, at optimum level of 38 mg Zn/kg. Although there was no influence of Zn-sulfate nor ZnPro on the Zn, Cu, Fe, concentration in the tibia (Table 4), Mn deposition in tibia of birds was affected by the Zn sources and levels under study. These responses were not clearly observed with the deposition of Mn in the liver. It was observed that the greatest Mn deposition occurred at the supplementation level of 76 mg ZnPro/kg, whereas no difference was observed in the Mn concentration in the tibia of birds fed Zn-sulfate.

When designing the current study, theory was postulated that dietary inclusion of Zn would affect antioxidant responses due to its role as cofactor for SOD. Liver of the birds fed diet supplemented with Zn, quadratically influenced SOD activity reaching a peak at the supplemental level of 44 mg/kg. However, effects of Zn supplementation were not noticed, on SOD activity in breast muscle. Such responses were consistent with our findings on tissue mineralization and could be correlated to Zn concentration in liver and breast muscle previously discussed. In the former organ, Zn deposition was stabilized at a dose of 38 mg/kg, whereas in breast muscle only a tendency was observed in Zn deposition. The Zn sources under study modulated the activity of GSH-Px in both liver and breast muscle. In both tissues, ZnPro fed birds exhibited a lower activity for the referred antioxidant enzyme compared with the group of birds fed Zn-sulfate diets. We believe that such responses are not associated with the organic Zn utilized in the current study, but rather to organic Se provided in ZnPro feeds. Glutathione peroxidase acts in synergy with SOD, and catalase in an antioxidant defense system and is modulated by Se status of the tissues. In the current study, Se was provided in ZnPro feeds as selenium yeast whereas Zn-sulfate fed birds were given sodium selenite as Se source. As detailed in [35], the utilization of dietary organic Se-Met for the synthesis of GSH-Px requires more steps than those from inorganic Se, which might reflect in a lower efficiency of organic Se utilization to produce selenoproteins. The referred authors observed a lower activity of GSH-Px in the blood of chicks fed selenium yeast compared with birds fed sodium selenite supplemented feeds.

Finally, the question of whether broiler chicken’s requirements for organic Zn are indeed lower than inorganic sources were addressed in the current paper by fitting curvilinear-plateau regression model to the collected performance data. As detailed in Table 6, ADG responses were optimized at approximately 30 and 19 mg supplemental Zn/kg feeds in chicks fed diets supplemented with Zn-sulfate and ZnPro, respectively, which corresponded to the concentration of 45 and 34 mg/kg diet. For inorganic Zn, the estimation of the findings is lower than the supplementation level of 74 mg/kg recommended by [36] for broilers, whereas for organic Zn the optimal levels barely differ between each other (34 vs. 33 mg Zn/kg). Irrespective of the Zn source, FCR was optimized at 16 mg Zn/kg feeds. Considering the Zn concentration in the basal feed, broiler chick requirements for Zn ranged between 31 and 45 mg/kg diet, depending on the sources of Zn and the performance response, which was previously explained, indicated that [9] recommendations are still adequate for the nutritional needs of fast-growing broilers The present study also confirmed the higher bioavailability of Zn and other organic minerals in comparison with inorganic sources and, most importantly, the agonistic interactions at intestinal and postabsorptive level can be minimized when trace minerals are entirely provided in organic form.

**Table 6:** Optimum dietary levels of Zn for broiler chicks considering sources individually or combined based on the interactive effects on growth performance

**Item**	Regression equations^1^	**Optimal zinc ** **supplementation mg/kg**	*p*-Value	**Coefficient of determination (R²)**
*Body weight*				
Inorganic	y = 486.3-0.00154 (22.67 - Zn)^2^	22.7	<0.01	0.528
Organic	y = 481.8-0.00262 (13.55 - Zn)^2^	13.6	<0.01	0.311
*Average Daily Gain*				
Inorganic	y = 35.2-0.00016 (30.32 - Zn)^2^	30.3	<0.01	0.565
Organic	y = 34.69-0.00076 (18.74 - Zn)^2^	18.7	<0.01	0.312
*Feed conversion ratio*				
Levels	y = 1400-0.0000000923 (16.35 - Zn)^2^	16.4	<0.01	0.197

^1^Regression equations obtained from fitting performance data to quadratic broken line regression model

## Conclusion

The study shows that FCR was optimized at 16 mg Zn/kg for both Zn sources, indicating a consistent effect of zinc on feed efficiency in broiler chickens. For ADG, the optimal levels were 30 mg Zn/kg for the inorganic source and 19 mg Zn/kg for the organic source, suggesting greater bioavailability and efficiency of the organic Zn. Additionally, inorganic Zn supplementation increased GSH-Px activity in the liver and breast muscle compared to the organic source, potentially enhancing antioxidant capacity in these tissues.

## Methods

### Ethics of animal care and use

All the animal care procedures involving animals were approved by the Institutional Animal Care and Use Committee of the Federal University of Viçosa, Viçosa, Minas Gerais, Brazil, before the beginning of the assay.

### Birds and husbandry

On d post hatch, 500 hundred 1-d-old, male Cobb, 500 chicks were obtained from a local commercial hatchery. From 1 to 7 d of age, during the pre-experimental period, birds were fed a pre-starter diet in mash form formulated to meet or exceed nutritional recommendations from [37] except for Zn, which was supplemented at 20 mg/kg as Zinc-sulphate (ZnSO_4_.H_2_O), to provide 50% of the Zn level recommended by [37]. The restriction in dietary Zn supply was adopted to avoid excessive Zn storage in chicks. Throughout the entire pre-experimental period, water and feed were provide ad libitum. On d 8 of age, chicks were housed in an environmentally controlled room and allotted into 49 cm × 27 cm × 33 cm (length x height x width) plastic cages with raised wire floors until the end of assay. Both experimental feeds and demineralized water were provided *ad libitum* throughout the 9-day experimental period (from 8 to 17 d of age). During the experimental period, the animals had no contact with any metal, avoiding any contamination or consumption of minerals not related with dietary treatments. Photoperiod consisted of 12 h natural light/12 h artificial light. Before the beginning of the assay at d 8 of age, all chicks were weighed and assigned to dietary treatment groups so that initial body weight (169.1 ± 1.6 g) was similar among experimental treatments. Broilers were randomly distributed in 10 treatments with 10 replicates and 5 chicks per cage, in a total of 100 cages, with each cage considered an experimental unit.

### Experimental diets and treatments

Experimental treatments consisted of a 2 × 5 fractional factorial arrangement to investigate the effect of 2 sources of Zn (organic and inorganic) and 5 supplemental Zn levels (0, 19, 38, 57, and 76 mg Zn/kg feed). The inorganic and organic Zn sources under study were Zn-sulphate (ZnSO_4_•H_2_O, 35.5% Zn) and Zn proteinate (ZnPro, 15% Zn - Bioplex^®^ - Alltech, Maringá, Brazil), respectively. A semi-purified basal diet, based on casein, albumin, corn, and dextrose (Table 7), was formulated to meet, or exceed nutritional requirements described by [37] for starter broilers, except for Zn. From the referred basal diet, 4 different diets were produced. The four diets differed in concentration and source of supplemental Zn (Zn-sulfate or ZnPro) as well as the trace mineral premix supplemented (organic trace mineral premix or inorganic trace mineral premix) as follows: (1) basal diet supplemented with organic trace mineral premix (ORG) without supplemental Zn; (2) ORG + 76 mg Zn/kg feed supplemented as ZnPro; (3) basal diet supplemented with inorganic trace mineral premix (INO) without supplementation of Zn; and (4) INO + 76 mg Zn/kg of feed supplemented as Zn-sulfate. Diets supplemented with organic or inorganic trace mineral premixes containing 0 and 76 mg Zn/kg as Zn-sulfate or ZnPro were mixed to produce five different inorganic or organic Zn levels: 0, 19, 38, 57, and 76 mg Zn/kg of feed. Sodium phytate was added to the semi-purified diet to simulate practical cereal-based diets. All experimental diets were supplemented with a commercial microbial phytase enzyme. All diets were analyzed for Zn concentration (Table 1) before the beginning of the assay as described by [38] (method 968.08). Overall, the analyzed concentrations of Zn were similar to the expected concentrations (Table 1).

**Table 7:** Proximate and nutritional composition of semi purified diets used in experimental period (8–17 days)

Ingredients, g/kg	
Corn	300
Albumin^1^	120
Starch	130
Dextrose	133
Casein^1^	40
Soybean protein isolate	40
Broken rice	80
Soybean oil	20
Cellulose^1^	40
Calcium carbonate^1^	16.95
Potassium phosphate^1^	14.85
Magnesium chloride^1^	6.50
Potassium chloride^1^	4.70
Choline chloride, 60%	3.70
Mixture of amino acids^2^	35.55
Micronutrients^3^	12.65
Microminerals^4^	2.00
Phytase^5^	0.1
*Calculated nutrients*	
AMEn, kcal/kg	3128
Crude protein^6^, %	22.58
*SID amino acids*,* %*	
Lysine, %	1.25
Methionine, %	0.55
Methionine + Cystine %	0.91
Threonine, %	0.83
Calcium^6^, %	0.77
Total P^6^, %	0.64
Available P, %	0.40
Zn^6^, mg/kg	15.00

^1^P.A purism reagent exceeds standard ACS specification in trace metals analysis

^2^0.03% L-lysine (79%); 0.27% L-arginine (98.5%); 0.40% L-glycine (98.5%); 0.85% L-alanine (99%); and 2.0% L-glutamic acid (99%). The amino acids alanine, glycine, and glutamic acid were added to maintain the ratio of essential nitrogen to total nitrogen at 0.50

^3^0.055% coccidiostat; 0.010% avilamycin; 0.030% BHT; 1.02% sodium phytate and 0.150% vitamin blend supplemented per kg of feed: vitamin A, 7500 IU; vitamin D_3_, 1900 IU; vitamin E, 28 IU; vitamin B_1_,2 mg; vitamin B_2_, 5 mg; vitamin B_6_, 1.2 mg; vitamin B_12_, 12 mcg; vitamin K, 1.5 mg; nicotinic acid, 0.03 mg; pantothenic acid, 0.01 mg; folic acid, 0.7 mg; and biotin, 0.07 mg

^5^Microbial phytase – 600 FTU/kg

^6^Value determined by analysis. Each value based on 10 replicates

### Performance and sample collection for Zn retention and tissue mineralization

Performance was evaluated from d 8 until d 17 of age. All the chicks and feed leftovers from each experimental unit were weighed to determine body weight (BW), and average daily feed intake (ADFI). From such data, average daily gain (ADG) and feed conversion ratio (FCR) were calculated. Mortality was also monitored, to adjust FCR when needed.

For Zn retention, during the performance assay, at d 13 of age, plastic trays were installed underneath the cages for total excreta collection throughout 4 days. Total excreta collection daily, weight, and frozen (−20 C) and at the end collection period were homogenized from each cage and a subsample were used for analysis of mineral content.

For broiler tissue mineralization evaluation, at d 18 of age, after a 8 h of fasting, one bird per cage (10 birds/treatment) was randomly selected and euthanized by cervical dislocation. A longitudinal incision was made in the abdominal cavity to collect the liver, and breast muscle, i.e., *pectoralis major*. The left tibia was also collected. The liver and muscle tissues were lyophilized for 72 h at − 80 °C under 800 mbar of pressure (Liobras– São Carlos, SP), grounded in a ball mill (Tecnal Equipamentos para Laboratório, TE-350, São Paulo, Brazil), and stored for further analysis of mineral content. After appropriate dilution, Zn, Cu, Mn, Fe contents were estimated by atomic absorption spectrophotometry (Spctr AA-800; Varian spectrometer, Harbor City, CA) in the Animal Nutrition Laboratory (Federal University of Viçosa, Viçosa, MG, Brazil) using [38] official method 968.08. Tibias were extracted for 4 h in a Soxhlet extractor (method 920.29 as described by [38] ground and analyzed for mineral concentration by the same procedures utilized in breast muscle and liver samples. Excreta samples were homogenized, dried in a forced-air oven at 65 °C for 48 h, grounded, and analyzed for mineral concentration.

### Antioxidant enzyme activity and lipid peroxidation

The activity of **s**uperoxide dismutase (SOD) and glutathione peroxidase (GSH-Px) was measured in *pectoralis major* samples (right side) collected after bird euthanasia and subsequently stored at −80 °C. The analysis was performed according to [39] using the kits of Randox Laboratories Ltda. (County Antrim, UK), through an automatic biochemical analyzer (Mindray BS-200E, Shenzhen Mindray Bio-Medical Electronics Co., Shenzhen, China) following the manufacturers’ guidelines. The extent of lipid peroxidation in breast muscle was estimated in terms of thiobarbituric acid reactive substances (TBARS), using malondialdehyde (MDA) as standard according to the method described by [40].

### Calculations and statistical analysis

The retention of Zn was calculated as follows: Zn retention (mg/bird) = [feed intake (g/bird) × feed Zn content (mg/g)] - [excreta output (g/bird) × excreta Zn content (mg/g)]. The Zn content in feed was based on calculated values using 10 replicates of individual analyses of each ingredient used in the diets and supplements. The Zn content of excreta was based on replicated analyses of excreta from each experimental unit. The Zn content in the basal diet was also considered in such equations.

Data were analyzed by the GLM procedure of SAS (SAS Institute Inc., Cary, NC) as a one-way ANOVA. The statistical model considered as the main effects the Zn levels, Zn sources, and interactions between the main effects. Each plastic cage containing 5 chicks was considered as an experimental unit. When the effect of Zn source was significant (*P* < 0.05), the means were compared using *F*-test, whereas the effects of Zn levels (*P* < 0.05) were compared using the Tukey´s multiple comparison test. Orthogonal polynomial contrast coefficients were used to determine linear and quadratic effects of increasing Zn supplementation levels (*P* < 0.05) on the investigated responses. The PROC NLIN and NLMIXED procedures of SAS were used to estimate the optimum Zn levels by organic and inorganic sources for BW, ADG, ADFI and FCR by a quadratic broken-line regression model [41] Statistical significance was accepted at *P* < 0.05 and a tendency at *P* < 0.10. Data are presented as least square means and SEM.

## Data Availability

The data set generated and/or analyzed during the current study is not publicly available since the data is a part of another study. The data is, however, available from the corresponding author on reasonable request.
